# Preparation of Pd Nanoparticles Stabilized by Modified Montmorillonite for Efficient Hydrodeoxygenation of Lignin-Derived Phenolic Compounds in Water

**DOI:** 10.3389/fchem.2022.961814

**Published:** 2022-08-05

**Authors:** Xuerong Wang, Chi Li, Xinyuan Guo, Zhichao Wang, Ruijing Cheng, Tianwei Xu, YiYing Li, Jinhui Wang, Huanjun Xu

**Affiliations:** ^1^ Key Laboratory of Child Cognition and Behavior Development of Hainan Province, Qiongtai Normal University, Haikou, China; ^2^ School of Science, Qiongtai Normal University, Haikou, China; ^3^ College of Basic Medicine and Life Sciences, Hainan Medical University, Haikou, China; ^4^ Department of Medicinal Chemistry and Natural Medicine Chemistry, College of Pharmacy, Harbin Medical University, Harbin, China

**Keywords:** lignin model compound, modified montmorillonite, hydrodeoxygenation, cycloalkanes, biofuels

## Abstract

Developing a new and efficient catalytic route for the production of alkanes by upgrading the aqueous phenolic biofuels still remains a challenge. Here, we designed and synthesized a bifunctional catalyst that uses natural montmorillonite (MMT) as support and combines metal active sites and Brӧnsted acid sites in the MMT *via* ion exchange and reduction roasting process. The catalytic activity of the as-synthesized Pd-MMT (H^+^) was evaluated by the hydrodeoxygenation (HDO) of a series of lignin-derived phenolic compounds in water. Our model reaction study reveals that the HDO of phenol undergoes an initial hydrogenation of aromatic rings to produce cyclohexanol and cyclohexanone, followed by the dehydration of cyclohexanol to provide intermediate cyclohexene and a final hydrogenation of cyclohexene to create a cyclohexane product. The combination of high metal catalytic activity and Brӧnsted acidity in Pd-MMT (H^+^) synergistically accelerated the HDO of phenol. Furthermore, good catalytic activity and recycling ability were also observed for other lignin-derived phenolic compounds.

## Introduction

The depletion of petroleum deposits and the increasing environmental concern regarding the burning of nonrenewable resources have diverted great attention toward recyclable resources. Biomass, therefore, is the best feedstock for the renewable production of biofuels, which could be a promising alternative to fossil fuels (Antar et al., 2021; Lee et al., 2021). Lignin is the second most abundant component of biomass, which is typically formed by methoxy-substituted phenylpropanoid units. After being processed by some technologies, it can be transformed into biofuels and special chemicals (Chio et al., 2019). The biofuels (most of those are phenols) decomposed from lignin are highly oxygenated, which, together with the unsaturated content of biofuels, leads to low oil quality problems, such as instability (Zakzeski et al., 2010; Saidi et al., 2014; Li et al., 2011; Ruiz et al., 2012; Jongerius et al., 2012; Ambursa et al., 2021).

Hydrodeoxygenation (HDO) is the most effective way to remove oxygen and some unsaturated content (Ambursa et al., 2021). Traditional catalysts for the HDO of biofuels are CoMo or NiMo sulfide, which have been widely used for industrial removal of sulfur, nitrogen, and oxygen from petroleum fuels (Zakzeski et al., 2010; Saidi et al., 2014; Li et al., 2011; Ruiz et al., 2012; Mortensena et al., 2011; Elliott, 2007; Liang et al., 2022). The disadvantage of the traditional catalysts includes sulfide contamination, coke formation, and deactivation caused by water. To overcome these shortcomings, researchers turned their attention to noble metals. The catalysts doped with noble metals showed better HDO activity and yield than traditional ones (Zhu et al., 2011; Lup et al., 2017; Lee et al., 2012). Meantime, various materials have been tested as support, such as ZrO_2_ (Lup et al., 2017), Al_2_O_3_-SiO_2_ (Lee et al., 2012), and Al_2_O_3_ (Bakhtyari et al., 2020). Recently, Zhao et al. (2009) reported an efficient difunctional catalytic system that combines carbon-supported noble metal catalysts and Brӧnsted acid (H_3_PO_4_) toward the HDO of lignin-derived phenolic fraction in water. This system has successfully hydrodeoxygenated phenol derivatives to produce cycloalkanes under a moderate condition (Zhao et al., 2009). However, this system used liquid H_3_PO_4_ as a Brӧnsted acid that is difficult to recover from the reaction mixture, harmful to the environment, and relatively corrosive to the reactor. Accordingly, some non-sulfide catalysts such as Pt/HY zeolite (Hong et al., 2010), Pt/HBeta (Zhu et al., 2011), Pt/C (Ohta et al., 2011), Ni-Nafion/SiO_2_ (Zhao et al., 2010), and metal nanocatalysts combined with Brӧnsted acidic ionic liquids (ILs) (Yan et al., 2010) have been recently evaluated for the HDO of phenols.

It is well known that clays are widespread, easily available, low-cost, and environment-friendly chemical substances with a layered structure (Nagendrappa, 2011). For example, MMT is one of the most common smectite clays and a promising naturally abundant and non-toxic reinforcing material which can be used as one of the components for food, medicine, cosmetic, and healthcare recipients (Ali et al., 2021; Vaculíková et al., 2021). The layers of clay possess net negative charge that is neutralized by cations such as Na^+^, K^+^, and Ca^2+^, which occupy the interlamellar space of MMT. The interlamellar cations can be easily replaced by other cations or other molecules with desirable functions (Vaculíková et al., 2021; Bokade and Yadav, 2011; Tsagkalias et al., 2021). Liu and coworkers presented a Ru-ionic liquids/montmorillonite catalyst system, which was found to be highly efficient in the hydrogenation of benzene (Miao et al., 2006). The electrostatic and coordination forces between the metal and catalytic support are very strong, which results in a very stable catalyst. In some organic reactions, clays have also been modified to act as solid acid catalysts (Motokura et al., 2006; Motokura et al., 2007; Motokura et al., 2012). Kiyotomi Kaneda described a novel synthetic method in which MMT was used as a solid acid in the addition reactions of 1,3-dicarbonyl compounds to alkenes, and the recovered catalyst could be reused at least seven times without appreciable loss of activity and selectivity (Motokura et al., 2006). More importantly, the acidic sites in MMT could interact with the O atom in ether bonds (Hechelski et al., 2018).

Therefore, we are intrigued with the idea that combining proton (H^+^)-modified MMT and noble metal forms a bifunctional catalyst, Pd-MMT (H^+^). MMT endows the catalyst with natural properties, and meanwhile, noble metal nanoparticles and H^+^ loaded within the MMT afford high catalytic activity to the Pd-MMT (H^+^). We envisage that such a bifunctional catalyst will be highly efficient in the HDO of lignin-derived phenolic fraction in water.

In this work, the idea is introduced to take Na^+^-montmorillonite (Na^+^-MMT) as the representative clay, which first exchanged protons to get H^+^-MMT and then doped with noble metal ions, that is, Pd^2+^, Ru^3+^, and Pt^2+^. The metal-supported MMTs were finally calcined to get the difunctional catalysts, denoted as Pd-MMT (H^+^), Ru-MMT (H^+^), and Pt-MMT (H^+^), respectively. Among them, Pd-MMT (H^+^) showed excellent catalytic activity towards the hydrogenation of aromatic ring and subsequent dehydration reactions, leading to a high HDO product of lignin-derived phenolic compounds.

## Experiment Section

### Materials

Unless otherwise stated, all chemicals in this work were commercially available and used without further purification. The chemicals were phenol (Sinopharm, >99% GC assay); anisole (Sinopharm, >98% GC assay); 4-n-propylphenol (TCI, >99% GC assay); 4-ethylguaiacol (Alfa Aesar, >98% GC assay); 4-hydroxy-3-methoxyphenylacetone (Alfa Aesar, >98% GC assay); guaiacol (J&K, >98% GC assay); 4-allyl-2-methoxyphenol (Alfa Aesar, >98% GC assay); diphenyl ether (J&K, >98% GC assay); cyclohexanol (Alfa Aesar, >99% GC assay); cyclohexanone (Alfa Aesar, >99% GC assay); hydrogen (Hainan Analytical Instrument Company, >99.999%); Na^+^-montmorillonite (Zhejiang sanding Co., Ltd.); Pd (NO_3_)_2_·2H_2_O; RuCl_3_ ; Pt (NO_3_)_2_ (Aladdin); phenethoxybenzene; and penzyl phenyl ether (Innochem, >97% GC assay).

### General Procedures for Aerobic Oxidation

The typical procedure was as follows: The desired amount of secondary alcohol substrate (0.5 mmol), Fe(NO_3_)_3_^**.**^9H_2_O (0.15 mmol), NaI (0.075 mmol), and DMSO (2 ml) were added into a 25-ml reaction bottle. Then, the mixture was degassed three times with the oxygen balloon; the reaction took place under 130 C for the desired time. After being acidified with 2 mol/L HCl (3 ml), the solution was extracted by ethyl acetate (5 ml) twice, and the organic phase was washed with saturated brine once and dried using Na_2_SO_4_. The combined organic phase was removed from the solvent by using a rotary evaporator. The desired product was obtained through column chromatography using ethyl acetate/petroleum ether as an eluent.

### Synthesis of Catalysts

H^+^-MMT was prepared according to a previously reported ion exchange procedure^[25]^. A mixture of Na^+^-MMT (3.0 g) and aqueous HCl (1 wt%, 200 ml) was stirred at 363 K for 24 h. A slurry was obtained after centrifugation. The slurry was washed with abundant distilled water repeatedly, then with ethanol, and then it was dried at 343 K in a vacuum overnight. The residual solid was finally grinded to obtain a gray powder, which was named H^+^-MMT.

Pd-MMT (H^+^) catalyst was prepared through a two-step procedure: First, 23.04 mg of palladium nitrate (Pd(NO_3_)_2_ 2H_2_O) was dissolved in distilled water (15 ml), and H^+^-MMT (1 g) was then added. The mixture was stirred at room temperature for 12 h, centrifuged, washed with water thoroughly, and finally washed with ethanol. The Pd^2+^-supported catalyst precursor was obtained after drying at 343 K in a vacuum overnight. In the second step, the catalyst precursor was reduced under H_2_ atmosphere at 473 K for 3 h to obtain Pd-MMT (H^+^). For comparison, Ru-MMT (H^+^) Pt-MMT (H^+^) and Pd-MMT (Na^+^) were also prepared using a similar procedure in which RuCl_3_ and Pt (NO_3_)_2_ were used as metal resources for Ru-MMT (H^+^) and Pt-MMT (H^+^), respectively and Na^+^-MMT was used as a support for Pd-MMT (Na^+^). The metal loading content in Pd-MMT (H^+^), Ru-MMT (H+), Pt-MMT (H^+^), and Pd-MMT (Na^+^) was 0.45, 0.42, 0.42, and 0.43 wt%, respectively, as determined by ICP-AES.

### Hydrodeoxygenation Reaction of Lignin Models

The HDO of lignin models was performed in a 15-ml stainless steel autoclave equipped with a magnetic stirrer. In a typical procedure, 2 mmol phenol, 40 mg catalyst, and 3 ml water are introduced into the reactor. The autoclave was first purged with H_2_ (0.2 MPa) three times to remove the air in it and then charged with 5 MPa H_2_ at room temperature. The autoclave was heated and maintained at the desired temperature (473 K or 493 K) for 1 h, and then stirred at 1,000 rpm. After a suitable reaction time, the reactor was cooled with ice water to quench the reaction. Conversion and yield were analyzed by using ethylbenzene as an internal standard. The organic phase was extracted from the mixture three times by ethyl acetate, and the combined organic phases were analyzed by an Agilent 6890 gas chromatograph equipped with an HP-INNOWax capillary column and an FID.

### Characterization

Powder X-ray diffraction (XRD) patterns were recorded on a Rigaku D/max-2500 X-ray diffractometer using Cu Kα radiation (*λ*= 0.15406 nm). The tube voltage was 40 kV, and the current was 200 mA. The X-ray photoelectron spectroscopy (XPS) data were obtained by using an ESCALab 220i-XL electron spectrometer from VG Scientific using 300 W Al Kα radiation. The base pressure was about 3 × 10^−9^ mbar. The binding energies were referenced to the C1s line at 284.9 eV from adventitious carbon. The specific surface area of the samples was determined by the N_2_ adsorption technique (Quantachrome Autosorb-1). The samples were degassed at 473 K for 3 h, and adsorption-desorption isotherms were measured at 77 K. The structural properties were characterized by transmission electron microscopy (TEM, JEOL JEM-2100F). The temperature programmed desorption (TPD) of ammonia was analyzed by using the Micromertitics AutoChem 2590 HP chemisorption analyzer. The catalysts were activated in He at 373 K for 1 h using a heating rate of 10°C min^−1^ from ambient temperature to 373 K. Ammonia was adsorbed by adding 10 vol% to the He carrier gas at 373 K. The sample was purged with He for 1.5 h in order to remove physisorbed molecules. For TPD of ammonia, the sample was heated in He at a rate of 10°C∙min^−1^ from 373 to 1033 K.

## Results and Discussion

**FIGURE 1:**
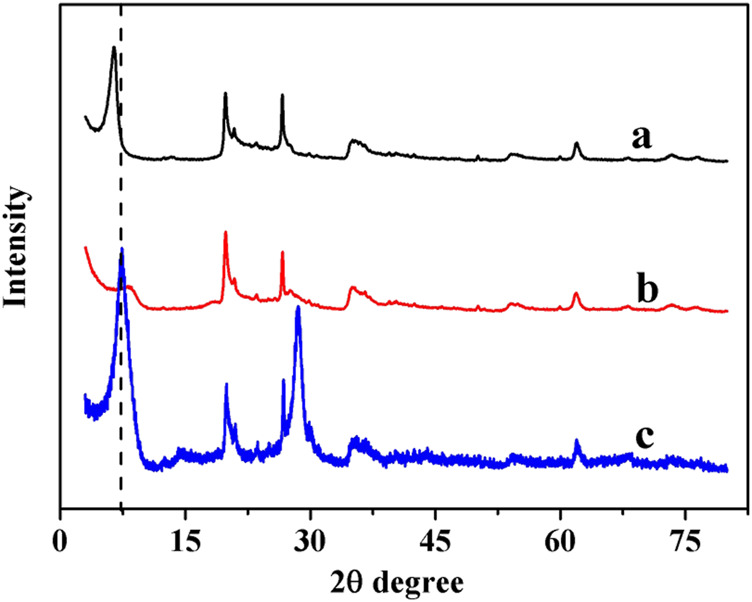
XRD patterns of **(A)** MMT (H^+^), **(B)** Pd-MMT (H^+^), and **(C)** MMT (Na^+^).

Figure 1 shows the XRD patterns of MMT (H^+^), Pd-MMT (H^+^), and MMT (Na^+^). The pattern of MMT (H^+^) became weak and shifted slightly to a lower value, implying that after being treated with HCl, the space between interlayers of MMT widened; however, the lamellar structure of MMT remained. No notable diffractive peaks of Pd or PdO appeared in the pattern of Pd-MMT (H^+^) compared with that of MMT (H^+^). However, it was observed that the intensity of the peak at 2θ = 9.6 was attenuated after Pd nanoparticles were loaded on the MMT (H^+^), indicating that the Pd nanoparticles were highly dispersed within the MMT (H^+^) support. All peaks of MMT (H^+^) were maintained after loading Pd nanoparticles, suggesting that the layered structure of MMT (H^+^) support was not destroyed, as confirmed by XRD measurement. The interlayer distance of the MMT (H^+^) was determined by subtracting the *c* dimension of the silicate sheet (9.6 Å) from the observed (001) values in the XRD spectrum (Motokura et al., 2012). Normally, the interlayer distance was enlarged by the added Pd nanoparticles that entered the layers of MMT (H^+^). However, the interlayer distance value was found to decrease in this work, as reflected by the increased 2θ value of the (001) peak (Figure 1). This can be ascribed to the lost water in MMT that was removed after the reduction roasting treatment, which was in accordance with Toshihide Baba’s research (Motokura et al., 2012).

**FIGURE 2:**
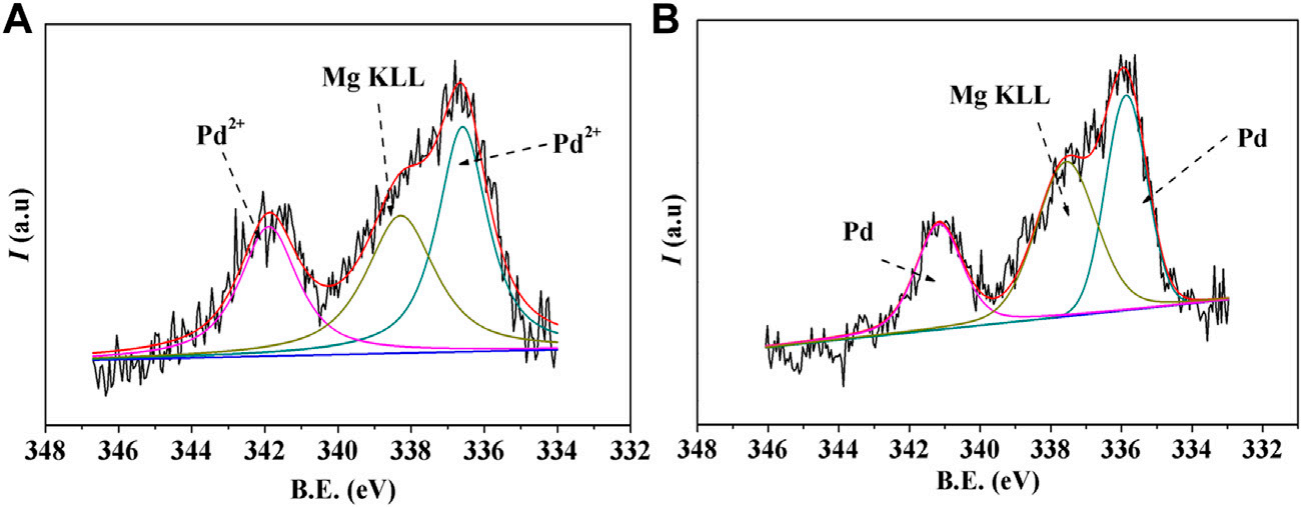
XPS spectra of the Pd 3d spectrum of Pd-MMT (H^+^) catalyst **(A)** before and **(B)** after reduction roasting.

The chemical states of Pd before and after reduction roasting were characterized by XPS, and the results are shown in Figure 2. In spite of the interference of the Mg in the natural MMT, the XPS spectrum could be resolved fairly well with two spin-orbit-split doublets from two chemically different Pd entities and the Mg (KLL) peak for the Pd-MMT (H^+^) before reduction roasting (Figure 2A). The peak binding energies of 336.5 eV (Pd 3d_5/2_) and 341.9 eV (Pd 3d_3/2_) correspond to the Pd^2+^ ion. After the reduction roasting, the peak binding energies were changed to 335.8 eV (Pd 3d_5/2_) and 341.1 eV (Pd 3d_3/2_), respectively, which illustrates that the Pd^2+^ ions have been successfully transformed into Pd^0^ nanoparticles.

**FIGURE 3:**
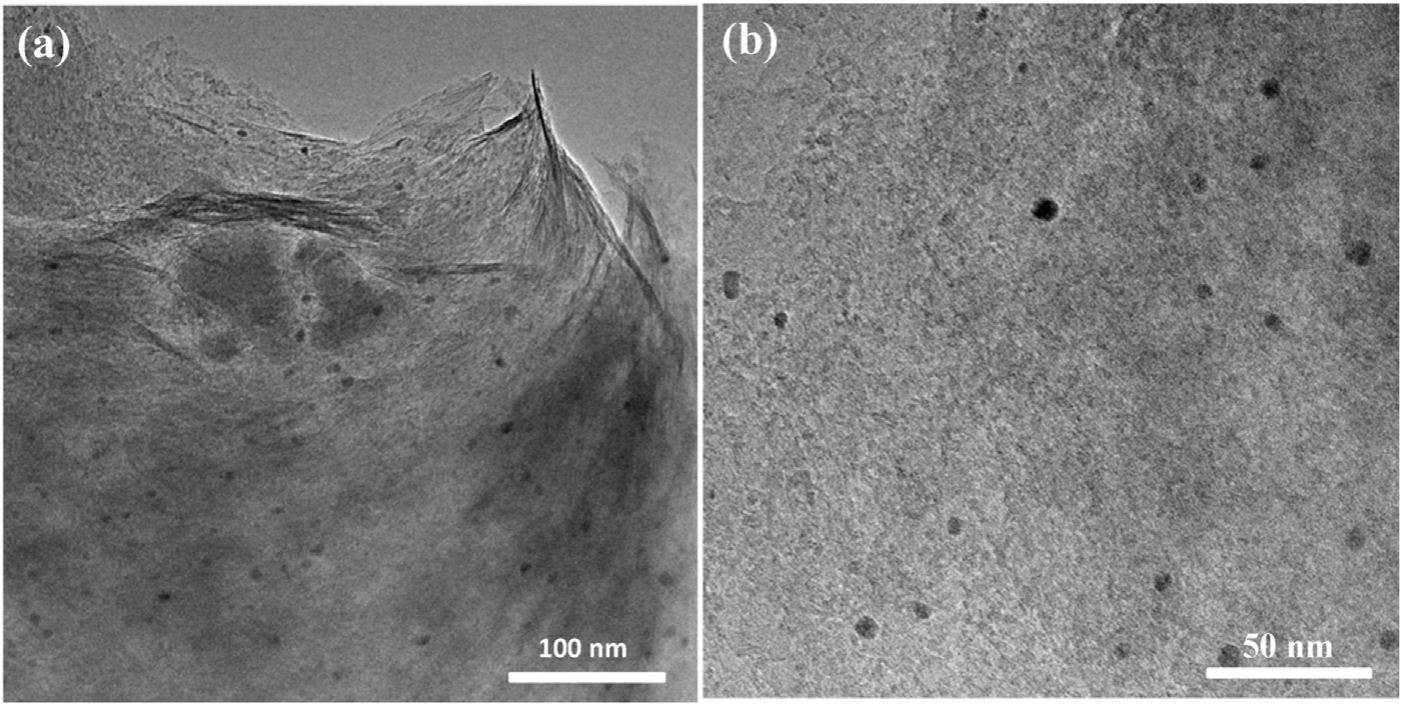
TEM images of Pd-MMT (H^+^).

The size and distribution of Pd nanoparticles have been observed by TEM images. It was shown that these spherical nanoparticles were uniformly dispersed within the MMT matrix with an average diameter of about 5–7 nm (Figure 3A). In the meantime, few Pd nanoparticles with a diameter close to 10 nm can also be observed, which was described as the presence of Pd nanoparticles on the outer surface of the MMT rather than inside the pores (Borah and Dutta, 2013).

**FIGURE 4:**
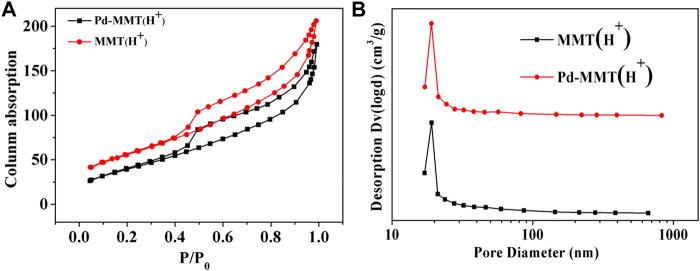
**(A)** N_2_ adsorption-desorption isotherms of MMT (H^+^) and Pd-MMT (H^+^); **(B)** Corresponding Barrett–Joyner–Halenda (BJH) pore size distribution curve determined from the desorption branch.

The porosity nature of MMT (Na^+^), MMT (H^+^), and Pd-MMT (H^+^) was measured by the N_2_ adsorption-desorption isotherm at 77 K. Figure 4A shows the typical type IV adsorption-desorption isotherm with a H_1_ hysteresis loop at P/Po = 0.4–0.9, indicative of a mesoporous structure (Xu et al., 2014). The pore size distribution curves calculated by the BJH method are shown in Figure 4B. The Brunauer–Emmett–Teller (BET) surface area, BJH pore sizes, and pore volumes were summarized in Table 1. The BET surface area of MMT (H^+^) and Pd-MMT (H^+^) (as determined from the desorption branch of the curve) was 200 and 148 m^2^ g^−1^, respectively, indicating that the BET surface area of MMT after being treated with HCl was significantly increased (Table 1 and Supplementary Figure S4). Meanwhile, after being treated with HCl, the BJH pore size distribution sharply increased to 19.071 nm, while the differential volumes versus pore diameter plot indicated relatively narrow pore size distributions.

**TABLE 1:** Textural properties of MMT (Na^+^), MMT (H^+^) and Pd-MMT (H^+^).

Sample	*SSA*_BET_ (m^2^ g^−1^)	Pore size (nm)	*V*_*total*_ (cm^3^ g^−1^)
MMT (Na^+^)	6	/	0.016
MMT (H^+^)	200	19.071	0.288
Pd-MMT (H^+^)	148	19.021	0.260

*SSA*_BET_, total BET specific surface area; *V*_*total*_, total pore volume.

It was notable that the BET surface area of Pd-MMT (H^+^) decreased, which might have resulted from the decrease in the distance between layers under high-temperature reduction roasting treatment. But after loading Pd nanoparticles, the isotherm shape was preserved and the BJH pore size distribution was not changed notably, indicating that the incorporated Pd nanoparticles did not block or alter the pore structure. The pore size was larger than 19 nm, which was ascribed to the dealumination of MMT during acidification (Pablo et al., 1998). The specific surface area and the pore diameter, as well as the pore size distribution of MMT, were changed after treatment with HCl. Those tuned properties of MMT make it act as an excellent “Host” for Pd^0^-nanoparticles.

The acidity of MMT (Na^+^) and MMT (H^+^) was estimated by NH_3_-TPD profiles (Supplementary Figure S5). MMT (Na^+^) only showed the strong peak that appeared at a high temperature of 450–650°C; meanwhile, the peaks of MMT (H^+^) appeared at 100–250°C and 450–650°C, respectively. It is evident that a new type of acidic site was produced in this acidification process (Ramesh et al., 2015). After acidification, there were still some Na^+^, K^+^, and Ca^2+^ ions remaining. The exchange of Pd^2+^ with MMT (H^+^) might be in an adsorption and exchange process, and as such, we predicted that there are some protons (H^+^) remaining in the Pd-MMT (H^+^).

### Catalytic Performance of Pd-MMT (H^+^)

**FIGURE 5:**
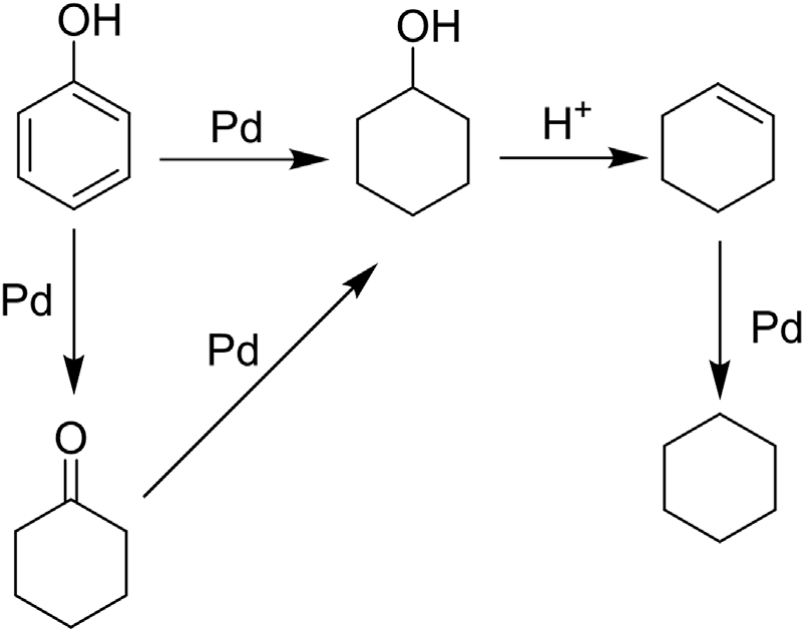
Proposed reaction pathway for the HDO of phenol over Pd-MMT (H^+^) to give cyclohexane.

To evaluate the catalytic performance of the synthesized Pd-MMT (H^+^), the HDO of a model compound, phenol, was chosen as the probe reaction. The reactions were performed at 473 K, 5.0 MPa H_2_, and the reaction time was 2 h. The experimental results are shown in Table 2. No products were obtained when Pd was absent in the catalyst (Table 2, entry 1). The appearance of Pd nanoparticles in Pd-MMT (Na^+^) led to a complete conversion of phenol to afford 38% cyclohexanol and 56% cyclohexanone products (Table 2, entry 2). In contrast, a 99% product of cyclohexane was observed when Pd-MMT (H^+^) was used as catalyst (Table 2, entry 3). These results indicated that the HDO of phenol may undergo two reduction steps: phenol may be first hydrogenated to cyclohexanone and cyclohexanol catalyzed by Pd nanoparticles. Herein, we cannot rule out the possibility that phenol was first reduced to cyclohexanone and the intermediate cyclohexanone was further hydrogenated to cyclohexanol. In the second step, cyclohexanol was further hydrogenated to produce cyclohexane that was catalyzed by the H^+^ acidity of Pd-MMT (H^+^) (Figure 5). This process was in accordance with Zhao’s report that phenol did not undergo direct hydrogenolysis to benzene and that only a combination of metal sites and Brӧnsted acid resulted in the HDO of phenolic compounds to alkanes (Zhao et al., 2010). Although carbon-supported platinum catalyst was also successful in catalyzing the HDO of phenols, the lack of hydronium ions required a high temperature of 553 K (Ohta et al., 2011). Obviously, the dehydration of cyclohexanol was accelerated by Brӧnsted acid sites on the Pd-MMT (H^+^) that significantly decreased the reaction temperature. The overall reaction pathway for the HDO of phenol to cyclohexane involves an initial Pd-catalyzed hydrogenation of the aromatic ring, a subsequent acid-catalyzed dehydration of cyclohexanol, and a final Pd-catalyzed hydrogenation of the cyclohexene to afford the cyclohexane product. A tentative mechanism for Pd-MMT (H^+^) hydrodeoxygenated lignin-derived phenolic compounds in water is as follows: The phenol was hydrogenated to cyclohexanone, and then the cyclohexanone was reduced to cyclohexanol. After that, the cyclohexanol was dehydrated to the cyclohexene, which was further reduced to cyclohexane (Figure 5).

**TABLE 2:** HDO of phenol in aqueous media^ab^.

Catalyst	Conversion (%)	Yield (%)
MMT (H^+^)	0	0 (cyclohexane)
Pd-MMT (Na^+^)	100	38 (cyclohexanol); 56 (cyclohexanone)
Pd-MMT (H^+^)	100	99 (cyclohexane)

a Reaction conditions: phenol 188 mg (2 mmol), catalyst 40 mg, H_2_O (3 ml), P(H_2_) = 5.0 MPa, T = 473 K, t = 2 h.

b Conversion was determined by gas chromatography (GC).

In addition to phenol, several important lignin-derived products were also successfully converted to their corresponding cycloalkanes (Table 3). Hydrogenation of these lignin-derived compounds leads to a variety of products. For instance, 4-propylphenol was transformed to n-propylcyclohexane in a high yield of 99% (Table 3, entry 1). Apart from Pd-MMT (H^+^), we attempted the HDO of 4-propylphenol with Pt-MMT (H^+^) and Ru-MMT (H^+^), respectively, and found that both Pt-MMT (H^+^) and Ru-MMT (H^+^) (Supplementary Figures S1, S2) can also efficiently catalyze the HDO reaction with a yield larger than 97% (Table 3, entry 2 and 3). However, anisole (Table 3, entry 4) and guaiacols (Table 3, entries 5–8) provided relatively low cycloalkane yield which varied from 50% to 73%, although a higher temperature (493 K) and a longer reaction time (8 h) were applied. This indicates that the methoxy group is unfavorable for the HDO to obtain a cycloalkane product.

**TABLE 3:** HDO of several lignin-derived compound models^a^.

Entry	Lignin models	Catalyst	Conversion (%)	Cycloalkanes yield (%)
1	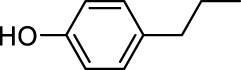	Pd-MMT (H^+^)	100	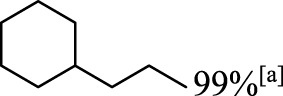 [Table-fn Tfn3]
2	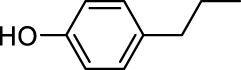	Pt-MMT (H^+^)	100	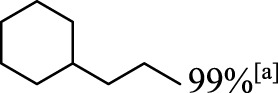 [Table-fn Tfn3]
3	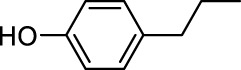	Ru-MMT (H^+^)	100	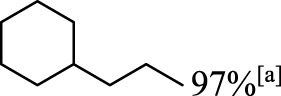 [Table-fn Tfn3]
4	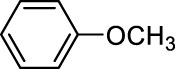	Pd-MMT (H^+^)	100	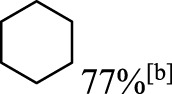 [Table-fn Tfn4]
5	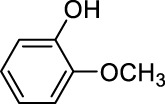	Pd-MMT (H^+^)	100	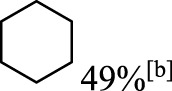 [Table-fn Tfn4]
6	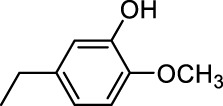	Pd-MMT (H^+^)	100	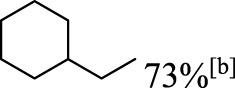 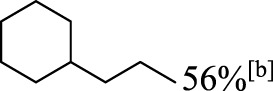 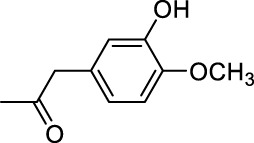 [Table-fn Tfn4]
7	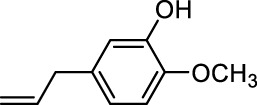	Pd-MMT (H^+^)	100	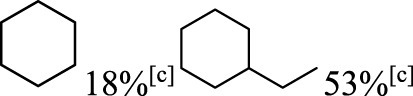 [Table-fn Tfn4]
8	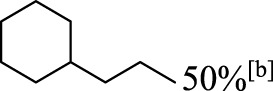	Pd-MMT (H^+^)	100	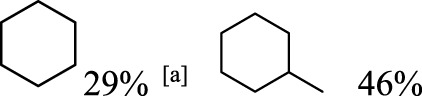 [Table-fn Tfn4]
9	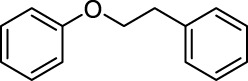	Pd-MMT (H^+^)	100	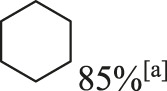 ^c^ ^c^
10	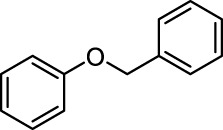	Pd-MMT (H^+^)	100	29%^a^ 46%
11	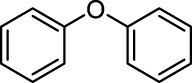	Pd-MMT (H^+^)	100	85%^a^

a Reaction conditions: phenols (2 mmol), catalyst (40 mg), H_2_O (3.0 ml), P(H_2_) = 5.0 MPa, T = 473 K; t = 2 h.

b T = 493 K, t = 8 h.

c T = 473 K, t = 4 h.

Furthermore, the bifunctional catalyst could be effectively applied in representational dimeric lignin models, such as phenethoxybenzene, benzyloxybenzene, and diphenyl ether (Table 3, entries 9–11). The total yield of cycloalkanes was between 71% and 85%. Among them, phenethoxybenzene with the most abundant linkage in lignin (β-O-4, 55% in lignin) was converted to 18% cyclohexane and 53% ethylcyclohexane at 473 K and 5 MPa H_2_ during 4 h reaction time. Benzyloxybenzene possessing α-O-4 linkage in lignin (8% in lignin) was also converted with a 29% yield of cyclohexane and a 46% yield of methylcyclohexane during a 2 h reaction. Diphenyl ether with 4-O-5 linkage was converted to cyclohexane with a yield of 85% at 473 K and 5 MPa H_2_. These results reveal that Pd-MMT (H^+^) not only easily depolymerized the linkages of representational lignin models but also further efficiently hydrogenated those to afford cycloalkanes.

The reusability of the Pd-MMT (H^+^) catalyst was investigated by the hydrogenation of 4-n-propylphenol. In each cycle, Pd-MMT (H^+^) was removed by centrifugation and then rinsed with 4-n-propylphenol. After drying, the catalyst was reused for the next run. The conversion of 4-n-propylphenol is 100, 100, 100, 98, and 98%, respectively, and the yield of n-propylcyclohexane is 99, 99, 99, 95, and 94%, respectively. Moreover, no Pd species were observed in the aqueous solution and organic solution detected by ICP. It was indicated that the Pd-MMT (H^+^) can be reused at least five times without significant loss of activity in the fifth run. Under the same reaction conditions, we performed the model reaction only using 1/5 dosage of Pd-MMT (H^+^) as a catalyst. A conversion rate of about 76% was reached. The reusability of this reaction was detected five times and the conversion was 75, 74, 72, 74, and 71%, respectively. These results indicate that Pd-MMT (H^+^) possesses good reusability for the HDO of lignin model compounds. The morphology of Pd-MMT (H^+^) was also examined by TEM observation after five recycles. It was found that the size of Pd nanoparticles slightly increased (Supplementary Figure S3), which might result in the slight decline in the conversion and yield of the HDO of the lignin model compound.

## Conclusion

In summary, we designed and prepared a bifunctional catalyst that combined Brӧnsted acid sites and metal active sites (Pd, Pt, and Ru) in montmorillonite (MMT) as support. The as-synthesized bifunctional Pd-MMT (H^+^) was characterized by XRD, XPS, TEM, and N_2_ adsorption-desorption isotherms. The catalytic performance of the Pd-MMT (H^+^) was evaluated by the hydrodeoxygenation (HDO) of lignin-derived phenolic compounds. As for the model reaction, the aromatic rings of phenol were hydrogenated to produce cyclohexanone and cyclohexanol catalyzed by Pd (0) nanoparticles, followed by the dehydration of cyclohexanol that was catalyzed by Brӧnsted acid in the Pd-MMT (H^+^) and further Pd (0)-catalyzed hydrogenation of cyclohexene to cyclohexane. The combination of Pd (0) nanoparticles and Brӧnsted acid in Pd-MMT (H^+^) accelerated the HDO process of phenol. This bifunctional Pd-MMT (H^+^) was also successfully applied to a series of lignin-derived phenolic compounds and exhibited good catalytic and recycle ability. This new approach may provide an efficient and economical route for upgrading lignin-derived phenolic oils to transportation biofuels.

## Data Availability

The original contributions presented in the study are included in the article/Supplementary Material; further inquiries can be directed to the corresponding authors.
