# Supplementary material for: Preparation of Pd Nanoparticles Stabilized by Modified Montmorillonite for Efficient Hydrodeoxygenation of Lignin-Derived Phenolic Compounds in Water
Source: Front Chem. 2022 Aug 5;10:961814. doi: 10.3389/fchem.2022.961814 (PMC9388857; doi:10.3389/fchem.2022.961814)
Supplement: Supplementary file 1 [file DataSheet1.docx]

Supplementary Material

**I. Materials**

Phenol (Sinopharm,>99% GC assay), Anisole (Sinopharm,>98% GC assay), 4-n-propylphenol (TCI,>99% GC assay), 4-ethylguaiacol (Alfa Aesar, >98% GC assay),4-hydroxy-3-methoxyphenylacetone(Alfa Aesar, >98% GC assay) , guaiacol(J&K, >98% GCassay),4-allyl-2-methoxyphenol (Alfa Aesar, >98% GC assay), diphenyl ether (J&K, >98% GC assay), cyclohexanol(Alfa Aesar, >99% GC assay), cyclohexatone(Alfa Aesar, >99% GC assay), hydrogen (Beijing Analytical Instrument Company, >99.999%). Na^+^-montmorillonite(Zhejiang sanding Co., Ltd), Pd(NO_3_)_2_•2H_2_O, RuCl_3_ and Pt(NO_3_)_2_ (Aladdin), phenethoxybenzene and penzyl phenyl ether(Innochem, >97% GCassay).

**II.TEM picture**


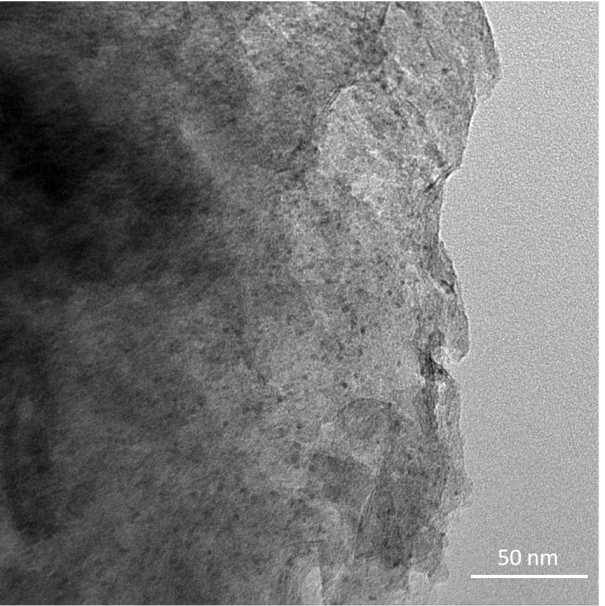


Figure S1: TEM image of Pt-MMT(H^+^)


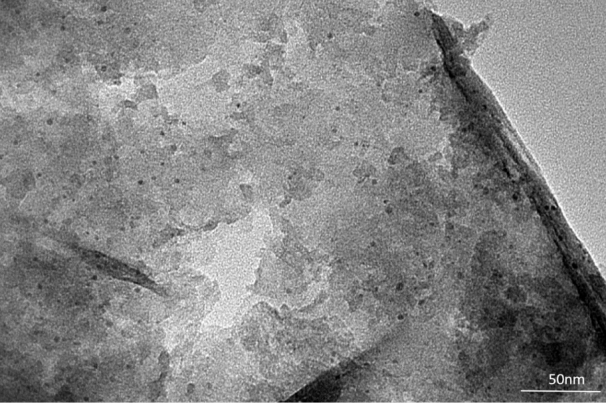


Figure S2：TEM image of Ru-MMT(H^+^)


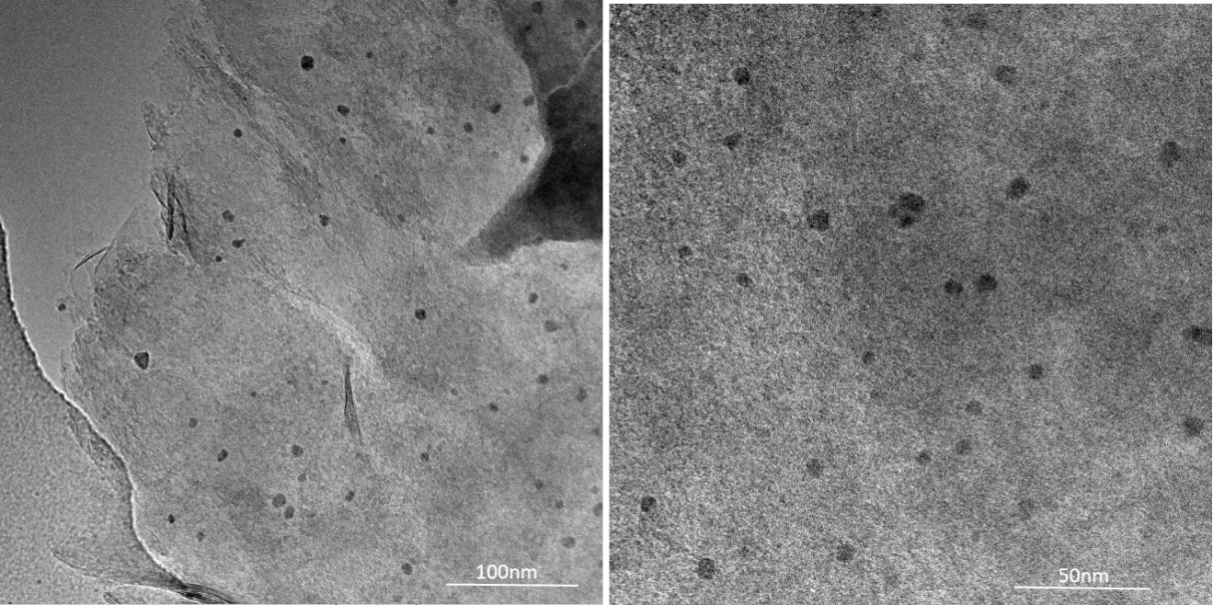


Figure S3: TEM image of Pd-MMT(H^+^) after being reused 5 times

**III. N_2_ adsorption-desorption isotherms of MMT(Na^+^)**

Figure S4: N_2_ adsorption-desorption isotherms of MMT(Na^+^)

**IV. NH_3_-TPD**

Figure S5: NH_3_-TPD of MMT(Na^+^) and MMT(H^+^).
